# Automatic Feature Selection for Imbalanced Echocardiogram Data Using Event-Based Self-Similarity

**DOI:** 10.3390/diagnostics15080976

**Published:** 2025-04-11

**Authors:** Huang-Nan Huang, Hong-Min Chen, Wei-Wen Lin, Rita Wiryasaputra, Yung-Cheng Chen, Yu-Huei Wang, Chao-Tung Yang

**Affiliations:** 1 Department of Smart Computing and Applied Mathematics, Tunghai University, Taichung 407224, Taiwan; 2 Cardiovascular Center, Taichung Veterans General Hospital, Taichung 407219, Taiwan; 3 Department of Post-Baccalaureate Medicine, National Chung Hsing University, Taichung 402202, Taiwan; 4 Department of Life Science, Tunghai University, Taichung 407224, Taiwan; 5 Department of Industrial Engineering and Enterprise Information, Tunghai University, Taichung 407224, Taiwan; rita.wiryasaputra@ukrida.ac.id; 6 Informatics Department, Krida Wacana University, Jakarta 11470, Indonesia; 7 Department of Computer Science, Tunghai University, Taichung 407224, Taiwan; 8 Research Center for Smart Sustainable Circular Economy, Tunghai University, Taichung 407224, Taiwan

**Keywords:** cardiovascular disease, classification, echocardiogram, feature selection, machine learning, voting ensemble

## Abstract

**Background and Objective:** Using echocardiogram data for cardiovascular disease (CVD) can lead to difficulties due to imbalanced datasets, leading to biased predictions. Machine learning models can enhance prognosis accuracy, but their effectiveness is influenced by optimal feature selection and robust classification techniques. This study introduces an event-based self-similarity approach to enhance automatic feature selection approach for imbalanced echocardiogram data. Critical features correlated with disease progression were identified by leveraging self-similarity patterns. This study used an echocardiogram dataset, visual presentations of high-frequency sound wave signals, and data of patients with heart disease who are treated using three treatment methods: catheter ablation, ventricular defibrillator, and drug control—over the course of three years. **Methods:** The dataset was classified into nine categories and Recursive Feature Elimination (RFE) was applied to identify the most relevant features, reducing model complexity while maintaining diagnostic accuracy. Machine learning classification models, including XGBoost and CATBoost, were trained and evaluated. **Results:** Both models achieved comparable accuracy values, 84.3% and 88.4%, respectively, under different normalization techniques. To further optimize performance, the models were combined into a voting ensemble, improving feature selection and predictive accuracy. Four essential features—age, aorta (AO), left ventricular (LV), and left atrium (LA)—were identified as critical for prognosis and were found in Random Forest (RF)-voting ensemble classifier. The results underscore the importance of feature selection techniques in handling imbalanced datasets, improving classification robustness, and reducing bias in automated prognosis systems. **Conclusions:** Our findings highlight the potential of machine learning-driven echocardiogram analysis to enhance patient care by providing accurate, data-driven assessments.

## 1. Introduction

Ensuring a healthy life is essential to support the realization of the United Nations’ Sustainable Development Goals (SDGs). A person’s lifestyle affects how healthy they are. Unhealthy eating patterns, smoking habits, lack of movement, excessive stress, high blood sugar, high cholesterol, and weather patterns can contribute to cardiovascular diseases (CVD), which is ranked the second-highest cause of mortality [1,2]. Bad weather can even tighten blood vessels and raise the chances of getting heart disease. As age increases, there is a linear increase in the risk of death. Older people are at a higher risk of cardiovascular and cerebrovascular diseases and are part of an age group that experiences a constant decline in heart health. Thus, the early detection of CVD is challenging. Additionally, between 1970 and 2020, the global population of seniors above the age of 60 years slightly increased from 10% to 13% [3], and the figure is projected to rise to 23% in 2050.

Invasive methods or non-invasive methods can be employed to diagnose heart disease [4]. Electrocardiography, phonocardiography, and echocardiography are non-invasive procedures commonly used to diagnose CVD. Regarding cardiac speed assessment, echocardiography facilitates a quick cardiac evaluation comprising the structure and heart function. Echocardiography features highly reliable in-resolution imaging and real-time feedback, affordability, and user-friendliness [4,5]. Although the non-invasive imaging detection approach has advantages, it also has drawbacks as it is lengthy, requires multiple evaluations thus increasing the complexity and user subjectivity, and is influenced by the physician’s experience. Moreover, the nature of a non-invasive approach requires a broader examination which creates varying complexities and medical opinions, even when conducted under standardized conditions [6,7]. To address these constraints, cutting-edge technology and artificial intelligence can usher in new atmospheres and breakthroughs to facilitate accurate, effective, and swift decision-making in healthcare.

A great interest has been focused on developing tools for predicting and managing many diseases with meaningful impacts on human health. The subset of artificial intelligence, machine learning, deals with endometriosis (EM) [8], breast cancer [9], lung cancer [10], arrhythmia [11], COVID-19 [12], Parkinson’s disease [13], chronic disease [14], acute chronic kidney disease [15,16], brain tumour [17],and CVD [1,3,4,18,19,20,21,22,23,24,25,26,27]. Table 1 shows some critical studies on CVD that utilize the methodology of machine learning and most studies used electrocardiogram data and a single dataset. The table contains the author’s name, the name of the classifier, experiment results, the number of features, the number of samples, and the name of the dataset used. Random Forest (RF) is used for general-purpose tasks; apart from its ability to handle mixed data, the model is also easy to use. Due to its high accuracy rates, Sumwiza [28] revealed that RF is a promising tool. Before implementing any feature selection approach, the accuracy of their proposed model reached 96%. This demonstrates the capability of the RF to handle dataset effectively. Even after using the correlation coefficient feature selection technique, the performance of the proposed model improved by approximately 3%. On the other hand, Wang [1] used data from patients in the coronary care units (CCU) and showed the performance of XGBoost outperformed Naïve Bayes, Logistics Regression, and Support Vector Machine (SVM) models. The accuracy score of XGBoost reached 66.3%, indicating that the XGBoost is a machine learning algorithm that offers specific characteristics: flexibility, efficiency in processing missing data, and accurate predictions even if there are weak prediction models. In other research, the performance results of XGBoost were not optimal. Sachdeva [19] used the UCI dataset of 299 heart failure patients and applied various classifiers like SVM, RF, Decision Tree (DT), and XGBoost to predict the survival of patients with cardiovascular diseases. They concluded that SVM worked better than the other classifiers and had an accuracy of 96.67%. LightGBM model offers a fast and efficient model, is memory-friendly, and is good with large datasets. Yang [29] proposed the LightGBM model based on an optional hyperparameter optimization framework validated with 4240 samples to mitigate the costs associated with the medical treatment of patients suffering from CVD. The XGBoost was also compared with the LightGBM. However, the performance of LightGBM achieved 97.8% on the area under the curve (AUC), outperforming other comparative models. The improved LightGBM model showed superior sensitivity, specificity, and accuracy. Data imbalance significantly affects data classification because it impacts model training convergence and generalization capabilities on unseen test data [30,31]. Regarding the early prevention of CVD, Boudali [32] evaluated the DT, RF, LightGBM, CATBoost, and XGBoost on the imbalanced dataset. The issue of imbalanced data is tackled by random under-sampling technique and LightGBM declares its ability to handle a large imbalanced dataset and emerge as the top performer with an accuracy rate of 77%. Among boosting approaches, the CATBoost classifier is also known as one of the most effective models with a high accuracy rate and a capability for versatility and adaptability [33]. The performance of CATBoost outperforms LightGBM and XGBoost, achieving an impressive accuracy rate of up to 98%. The CATBoost’s advantages in dealing with classified variables and producing reliable results were utilized by Wei [34] in CVD risk prediction. Their proposed CATBoost model reached its accuracy at 86.30%, thus, the robustness of CATBoost is suitable to support physicians in potential risks due to reducing the misdiagnosis rate. Individual and voting techniques can also be used to solve CVD issues; most previous studies used a single predictive model. Sen [35] proposed a soft voting meta classifier composed of CATBoost, XGBoost, LightGBM, Gaussian Naive Bayes, and RF, which was then trained with 918 samples of CVD electrocardiogram and 11 features. The accuracy of the soft voting ensemble model achieved 91.85% and a 0.9344 AUC score, indicating strong predictive performance and highly effective in distinguishing between CVD-positive and CVD-negative cases. The work presented in [36,37] used hard voting ensemble approach with more than 10 parameters. The performance of their proposed model reached an accuracy of 90%.

The result of an echocardiography procedure is an ultrasound image, also known as an echocardiogram in the US, which is the visual representation of the high-frequency sound wave signals propagating through an object. An echocardiogram and clinical dataset contain critical features that influence the decision-making. Many irrelevant and redundant features in a dataset cause model performance to decrease because of over-fitting and dimensionality issues. The number of features in any dataset can be reduced by using different approaches [38]. The Recursive Feature Elimination (RFE) algorithm for feature selection is commonly used in machine learning [39]. By iteratively selecting the most critical features and eliminating the weakest ones, this approach can improve the model’s performance and remove irrelevant or redundant features that may cause overfitting [40]. The resulting feature’s importance ranking can help to identify the most informative feature and optimize the model accuracy while reducing the computational complexity and training time. This technique is beneficial for analyzing complex and high-dimensional datasets, such as medical images or signals, where selecting relevant features can be challenging due to the large number of potential variables [9,13].

The novel contribution of this research work is as follows:

- Addresses the issue of imbalanced echocardiogram datasets by integrating the RFE feature selection technique to identify the potential features of CVD, reducing model complexity while maintaining accuracy.
- Demonstrates the effectiveness of ensemble voting strategies in enhancing prediction robustness, combining classifiers to improve feature selection and diagnosis accuracy.
- Proposes a robust classification pipeline incorporating XGBoost, RF, LightGBM, CATBoost, and ensemble learning to mitigate bias and improve predictive performance.
- Provides a clinically relevant feature subset facilitating better decision-making in healthcare

The proposed study aims to assist clinical decision-making with an automated prognosis system with effective prediction models and automated feature selection in the context of imbalanced datasets. The paper is organized as follows: Section 1 presents a comprehensive review of cardiac ultrasound and machine learning. Materials and methods are presented in Section 2 as part of the background research. The Section 3 describes the results. The Section 4 explains the experiment processing. The research conclusions and future work are given in the Section 5.

## 2. Materials and Methods

**Figure 1:**
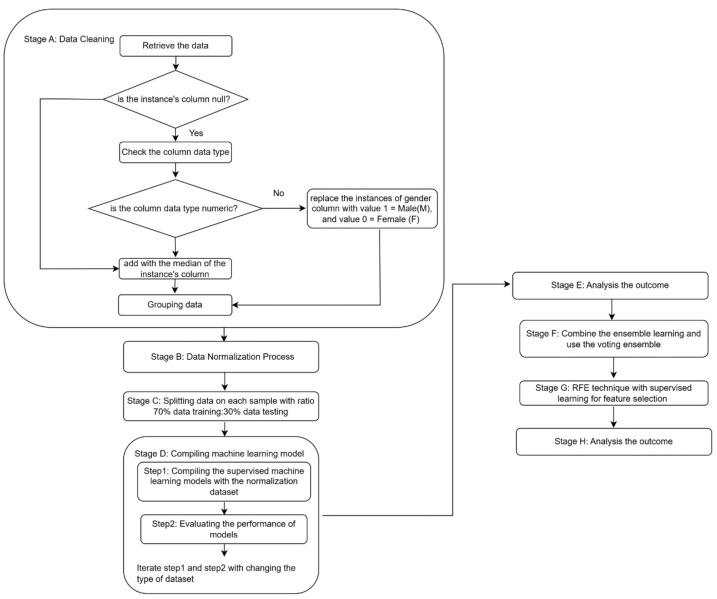
Block diagram.

This section describes the data collection, the classification methods incorporating the supervised learning method, and the feature selection method. Four supervised methods, XGBoost, RF, LightGBM, and CATBoost, were evaluated in the model-construction phase. The supervised technique entails the utilization of predetermined independent variables as features or risk factors. That dependent variable is the response outcome data for a model to predict novel instances once trained. It comprises independent input variables and their dependent variables outputs. The CVD early diagnosis approach uses ultrasound heart exam equipped with machine learning methods. Figure 1 shows the research block diagram, and the pseudocode of the proposed model is shown in Appendix A.

### 2.1. Data Source

**Figure 2:**
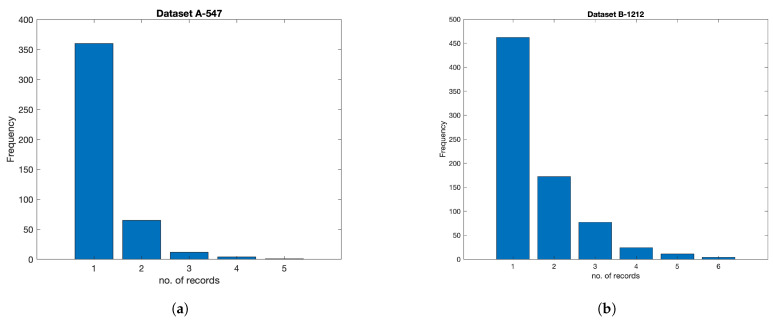
Frequency of sample A and sample B. (**a**) Frequency of sample A, (**b**) Frequency of sample B.

Taichung Veterans General Cardiologists provided two sample datasets: sample A consists of 547 records, and sample B consists of 1212 records; both datasets are taken from the diagnosis result and treatment section and the patients’ historical visitations section over three years. During three years, a patient may have several records with information about his diagnosis, visitation, treatment, and the result of a series of cardiac ultrasound tracking. The simple matching method and event-based self-similarity approach were used to find corresponding patients based on clinical characteristics such as age, gender, primary diagnosis, and medical histories to ensure these patients had similar ultrasound report numbers and clinical backgrounds. Dataset sample B contains all the records over three years and is employed to classify the task. To understand the impact of dataset size on machine learning model performance, dataset sample A was created. Dataset sample A is a subset of dataset sample B. These are new records of patients who did a series of tracking cardiac ultrasounds because their heart’s physiological condition worsened compared to previously recorded data. There are 745 records with CVD and 561 records without CVD. A patient may have several records, whereas the average is 1–2 records per patient. Figure 2a shows the frequencies of distribution patients—from one to five—corresponding with the number of records in sample A, with details as follows: 360, 65, 12, 4, 1, respectively. Figure 2b shows the frequencies of distribution patients corresponding with the number of records in sample B, with details as follows: 462, 172, 77, 24, 11, 4, respectively.

Heart disease treatments are performed through cardiac catheter ablation, ventricular defibrillator, and drug control. Regarding the diagnosis result categories, the dataset collection is organized into several statuses: died, one-time or more than one-time follow-up in cardiology and surgery, and no follow-up. Table 2 shows the grouping—Group 11, Group 13, Group 14, Group 21, Group 23, Group 24, Group 31, Group 33, and Group 34—based on treatments and diagnosis cohort.

Group 11 corresponds to patients who received cardiac catheter ablation treatment and were recorded as deceased. Group 13 corresponds to patients who received cardiac catheter ablation treatment and had more than one-time follow-up visits after treatment. Group 14 corresponds to patients who received cardiac catheter ablation treatment but did not return for continued treatment.

Group 21 corresponds to patients who received ventricular defibrillator treatment and were recorded as deceased. Group 23 corresponds to patients who received ventricular defibrillator treatment and continued with more than one-time follow-up visits after treatment. Group 24 corresponds to patients who received ventricular defibrillator treatment but did not return for continued treatment.

Group 31 corresponds to patients who underwent drug control treatment and were recorded as deceased. Group 33 corresponds to patients who undertook drug control treatment and proceeded with more than one-time follow-up visits after treatment. Group 34 corresponds to patients who underwent drug control treatment but did not return for further treatment.

Table 3 and Table 4 show the distribution of patients across diagnosis and treatment categories, which was imbalanced and highlights that the most significant number of patients belong to Group 23, which received ventricular defibrillator treatment and ongoing follow-up visits for diagnosis. The term imbalanced datasets describes when most classes outnumber the minority classes [41]. From sample A, 40.21% of the records were for patients who had a ventricular defibrillator treatment and were re-visited to follow up on their treatment (220 records). From sample B, 41.09% of the records were for patients who had a ventricular defibrillator treatment and received ongoing follow-up visits for diagnosis (498 records).

Table 5 shows the descriptive statistics result of Group 23 in sample A. It shows the age range of 26 years old to 96 years old in which the mean is 77 years old; the mean of Left Ventricular (LV) is 46.8 mm; the mean of Ventricular Septum (VS) is 13 mm; the mean of Left Ventricle Posterior Wall (LVPW) is 12.6 mm; the mean of left atrium (LA) is 46.5 mm; the mean of aorta (AO) is 36.2 mm; the mean of TR mean pressure gradient (TR_PG_Mean) is 24.2 mmHG; and the mean of Left Ventricular Ejection Fraction (LVEF) is 56.

**Figure 3:**
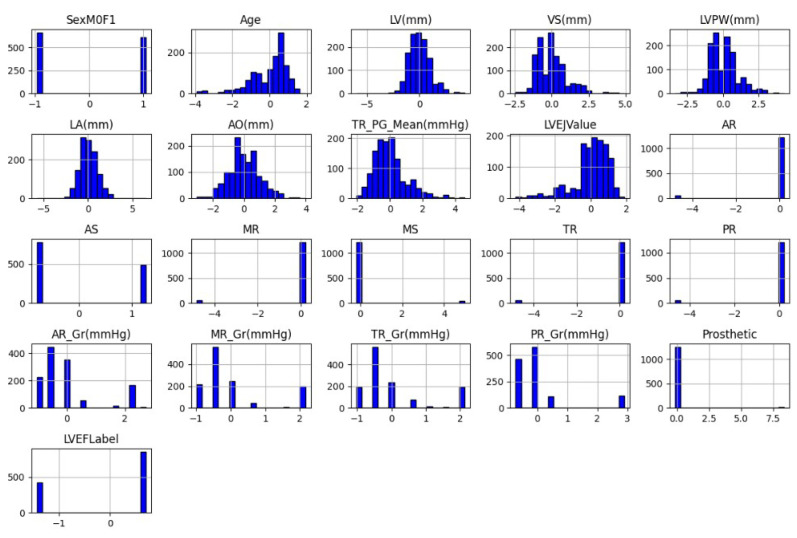
Histogram for the continous attributes.

A preview of the data distribution on continuous attributes is visualized through a histogram as shown in Figure 3. It can be noted that some continuous numerical features, such as age, LV, VS, AO, LVPW, and LA, show a bell-shaped distribution, indicating that it is beneficial for statistical modeling and machine learning algorithms.

To assess the model’s generalizability and robustness, the dataset was divided into training and testing sets, with proportionate allocations of 70% and 30%, respectively. The distribution of training and testing data composition is applied to samples A and B.

### 2.2. Random Forest (RF)

This classifier builds an ensemble of *M* decision trees and combines their outputs for the final prediction [42,43], represented as:

(1) $$m_{M,n}\left(x;Q_{1},\ldots ,Q_{m},\partial_{n}\right)=\frac{1}{M}\sum_{i=1}^{m}m_{n}\left(x;Q_{i},\partial_{n}\right)$$

where *M* can be any size but is limited to computing resources. Considering the *i*-th tree in a cluster of trees, the predicted value at every query point *x* is denoted by $m_{n}\left(x;Q_{i},\partial_{n}\right)$, where $Q_{1},\ldots ,Q_{m}$ is the independent random variables and $\partial_{n}$ is the training time.

Each tree in the forest is built from a random subset of the training data and a random subset of features to enhance model robustness and avoid overfitting. The RF classifier has the hyperparameter as follows: the number of estimators, the maximum depth for each tree, the number of minimal samples that is needed to split the nodes, the minimal number of samples of leaf.

### 2.3. XGBoost

XGBoost is a constructed tree ensemble algorithm under the Gradient Boosting Decision Tree (GBDT) framework. It combines the advantages of bagging and boosting, namely training loss and regularization, to prevent overfitting of the model, and it can be used in the regression. For example, Gradient Boosting refits the errors of all previous weak learners by adding a new weak learner iteratively, in which the final accuracy will be higher than what would be achieved using a single learner. XGBoost retains this feature so that each tree is related and keeps the original model unchanged in each operation. It then adds a new function to the model so that the tree generated later can correct the errors of the previous tree. XGBoost uses random feature extraction when generating trees; hence, not all features will be used in every decision-making process during tree generation. Each decision tree calculates the feature and threshold with the best branch effect and completes the split construction. The hyperparameter of XGBoost is as follows: the number of boosting rounds, known as the estimator number, the number of learning rates, the maximum depth of the tree, and the minimum weight of the child node.

In Gradient Boosting [44], each new model $f_{m}\left(x\right)$ is added to correct the errors of the previous models. The model is built iteratively by minimizing the loss function $L(y,\hat{y})$ over all training instances. The prediction at stage *m* is given by [43] :

(2) $${\hat{y}}^{\left(m\right)}={\hat{y}}^{\left(m-1\right)}+\eta f_{m}\left(x\right)$$

where: ${\hat{y}}^{\left(m\right)}$ is the prediction at stage *m*, η is learning rate, $f_{m}\left(x\right)$ is the *m*-th weak learner (a decision tree in this case)

The objective function for XGBoost includes a regularized loss function to balance model complexity and predictive accuracy [44]:

(3) $$L=\sum_{i=1}^{n}L\left(y_{i},{\hat{y}}_{i}\right)+\sum_{k=1}^{K}\Omega \left(f_{k}\right)$$

where $L(y_{i},{\hat{y}}_{i})$ is training loss function such as mean squared error for regression, $\Omega (f_{k})$ is the regularization term for tree complexity, with *T* as the number of leaves, γ controls the number of leaves, and λ controls the leaf weights. Total number of boosting rounds or estimators *M*. Each round introduces a new tree to improve the model. Learning rate η controls the contribution of each tree. A smaller learning rate requires more rounds. Maximum depth $d_{\max}$ of each tree, controlling the model’s complexity and overfitting. Minimum sum of instance weights required in a child node $w_{\min}$, helping prevent overfitting on small samples.

### 2.4. LightGBM

LightGBM is a Gradient Boosting Decision Tree (GBDT) model optimized for high efficiency and low memory usage. It iteratively adds decision trees to the ensemble [45] and uses a histogram-based algorithm which reduces memory usage and improves speed by binning continuous features into discrete intervals [32], as demonstrated in Equation (4).

(4) $$\mathrm{bin}\left(x_{i,j}\right)=\left\lfloor \frac{x_{i,j}-\min\left(X_{j}\right)}{\max\left(X_{j}\right)-\min\left(X_{j}\right)}\times B\right\rfloor$$

Unlike Level-wise, which splits nodes at the same level in each step, LightGBM grows trees Leaf-wise. This approach allows each split to focus on the leaf with the highest error reduction. While it can improve accuracy, it may lead to overfitting, especially on small datasets. LightGBM introduces a maximum depth for leaves, $d_{\max}$, which limits how deep the tree can grow to prevent overfitting. This parameter provides a balance between model complexity and overfitting. LightGBM achieves high training speed, up to 10 times faster than traditional GBDT.

### 2.5. CATBoost

CATBoost is a machine learning algorithm developed by Yandex that optimizes gradient boosting specifically for datasets with categorical features [33,34]. It implements ordered boosting and dynamic partitioning to handle categorical variables more effectively, reducing overfitting and enhancing accuracy. Traditional gradient boosting often introduces overfitting, especially in datasets with categorical features. CATBoost mitigates this with ordered boosting, which ensures that at each boosting iteration, only the past data is used to calculate predictions, preventing target leakage and overfitting. CATBoost applies dynamic encoding to categorical features using feature combinations. It encodes categorical values by averaging target values for subsets, effectively managing categorical features in high-dimensional spaces. The prediction equation [17] is shown in Equation (5):

(5) $${\hat{y}}^{\left(m\right)}={\hat{y}}^{\left(m-1\right)}+\eta f_{m}\left(x\right)$$

where η is the learning rate, $f_{m}\left(x\right)$ is the decision tree added at iteration *m*. The number of iterations *m* is a hyperparameter controlling how many trees are added to the model, which affects accuracy. Higher values of iterations may improve the accuracy model, but increase the computational cost. Learning Rate η controls the contribution of each tree in the boosting process.

### 2.6. Normalization Techniques

Raw dataset is the initial, unprocessed data directly obtained from the source. Raw datasets often contain features with varying scales, which can lead to biased model performance if not appropriately scaled, especially for algorithms sensitive to feature magnitude, such as the k-nearest neighbors, SVM. The Z-score normalization transforms the features by removing the mean and scaling to unit variance, resulting in a distribution with a mean of 0 and a standard deviation of 1 for each feature. This is useful when features in the dataset are normally distributed and when we want to center data around zero which can improve model convergence during training. The transformation formula [37,46] is:

(6) $$X_{\mathrm{scaled}}=\frac{X-\mu }{\sigma }$$

where *X* is the feature value, μ is the mean, and σ is the standard deviation. The absolute maximum normalization approach scales each feature by its maximum absolute value, transforming the data to lie within the range [−1, 1]. This scaler preserves sparsity, making it suitable for sparse datasets, and is often used when feature values have no fixed bounds but differ in magnitude. Each of these scaling methods serves distinct purposes, depending on the dataset’s characteristics and the requirements of the chosen machine learning model.

### 2.7. Recursive Feature Elimination (RFE)

RFE is a wrapper-based feature selection method that recursively removes features and builds a model on the remaining features [47]. Then it ranks the features by importance and eliminates the least important ones. The resulting feature’s importance ranking can help to identify the most informative features and optimize the model accuracy while reducing the computational complexity and training time. The process is repeated until a predetermined number of features are reached. Based on this circumstance, the most critical features with the highest impact on the target variable are identified. The most critical features can be used for better model performance and efficiency. This approach can improve the model’s performance and remove irrelevant or redundant features that may cause overfitting. This technique is beneficial for analyzing complex and high-dimensional datasets such as medical images or signals, where selecting relevant features can be challenging due to the large number of potential variables.

## 3. Results

Table 6 shows the performance of various supervised learning models (XGBoost, RF, LightGBM, and CATBoost) in two distinct samples, sample A and sample B, each evaluated under three scaling conditions: raw dataset, Z-score normalization, and absolute maximum normalization in both cases. A significant accuracy gap is evident between sample A and sample B across all models, with sample B achieving much higher accuracy values. For instance, the CATBoost model shows an accuracy of 0.394 across all scaling methods in sample A, but this accuracy jumps to 0.884 in sample B under the same conditions. This discrepancy points to an imbalance in data quality or sample representativeness, leading to varying model performance depending on sample characteristics. The accuracy gap is particularly noteworthy in cases where Z-score normalization marginally improves model accuracy, especially in sample B. For example, RF’s accuracy in sample B improves from 0.851 (Raw dataset) to 0.868 (Z-score normalization), suggesting that scaling techniques can mitigate some of the effects of data imbalance but may not entirely address underlying distributional differences between samples. This unbalanced performance indicates that the dataset’s inherent characteristics, such as feature distributions and class proportions, may differ significantly between sample A and sample B. This imbalance could lead to biased predictions, particularly in scenarios where models trained on sample A might underperform due to a lack of feature scaling or sample diversity, thereby limiting the generalizability of the supervised learning models.

In sample A, the highest accuracy percentage of 41.2% was reached using the LightGBM approach with Z-score normalization, while in another case, its accuracy was dominant at 87.6%. Based on these results, LightGBM is an effective model where the performance may be limited by the preprocessing method and the dataset’s characteristics. The difference in the feature distribution in the dataset is a factor that may cause significant differences in accuracy values. The sensitivity of LightGBM to hyperparameters and data complexity means that the Z-score normalization process may not be optimal for this particular case. In sample B, the supervised approaches had an accuracy percentage of more than 80% and showed exemplary achievements. The superior performances demonstrate their robustness when trained on a larger dataset containing more representative and diverse features, allowing supervised models to generalize better. Increasing the amount of data significantly improves the accuracy of supervised learning, with an improvement of up to 40%. This result is consistent with those of the previous study [48]. Although the models applied normalization and unnormalization approaches, the difference in the performance was insignificant. As shown in Table 6, the XGBoost and the CATBoost models achieved accuracy values of 84.3% and 88.4%, respectively. Applying the normalization approach to data did not change the accuracy values significantly. In sample B, the CATBoost model was the most popular.

Table 7 and Table 8 show the evaluation metrics for the proposed machine learning models on training and testing datasets. Overall, all models achieved very high performance on the training set, with precision, recall, and F1-score exceeding 0.99 in most cases. However, the RF experienced a lack of variability or potential overfitting. In the test set, the models’ performances decreased slightly. XGBoost and LightGBM show a moderate decrease, but their scores are still relatively high, indicating a good balance between learning and generalization. CATBoost seems to generalize the best, as its training and testing scores remain very close. CATBoost maintains high consistency, making it a reliable choice for predicting CVD prognosis.

Table 9 shows the accuracy scores obtained from 10-fold cross-validation for different machine learning models that predict CVD prognosis. The accuracy values vary slightly across different folds. However, they remain within a reasonable range. Some variations are expected due to each fold’s different training and validation splits. LightGBM demonstrated strong performance with an accuracy mean of 0.892, followed closely by XGBoost (0.881), CATBoost (0.866), and RF (0.853). The standard deviation of accuracies (XGBoost: 0.023, RF: 0.022, LightGBM: 0.022, CATBoost: 0.020) indicated stable and consistent model performance across folds. The performance of LightGBM often reach its accuracy above 0.90; its achievement was consistent across most folds. LightGBM achieves the highest accuracy by multiple folds, indicating strong predictive performance. On the other hand, RF had a lower accuracy score than XGBoost and LightGBM, indicating it might not generalize as well for the proposed dataset. XGBoost achieved its accuracy values close to LightGBM in multiple folds, indicating it performed well. CATBoost provides moderate results but does not outperform LightGBM or XGBoost. Its accuracy values were slightly lower in some folds than XGBoost.

**Figure 4:**
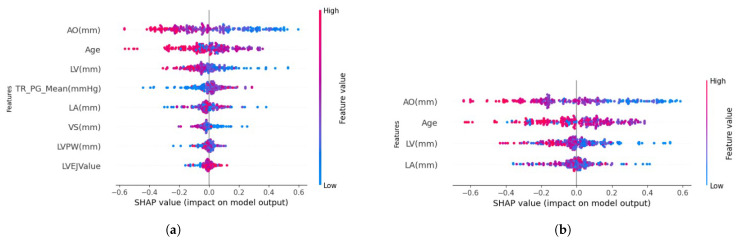
The ranked of features in the SHAP plot. (**a**) SHAP of XGBoost ensemble with 8 features, (**b**) SHAP of RF-Voting ensemble with 4 features.

Table 10 shows the result of implementing the RFE technique with the voting ensemble-supervised learning approaches. The accuracy of the ensemble technique (A) is analyzed across feature sets (F) to optimize the performance of the model. Leveraging the models’ performance with the voting ensemble-supervised learning framework, mainly RF, reached a accuracy of 96.9%. The highest achievement of the RF-voting ensemble model was constructed with four features. Four dominant features—age, AO, LV, and LA—influence the model’s performance. As age increases, the LA value becomes higher. Table 10 highlights that many features do not determine the model performance. The accuracy score of the predictive LightGBM-XGBoost combination with 15 features was similar to that gained by the voting ensemble-XGBoost combination with 8 features. The relationship between the number of features and accuracy is not always proportional or inversely proportional. Therefore, the SHapley Additive Explanations (SHAP) plot was employed to analyze how the echocardiogram features affect disease prediction. The SHAP plot ranks the features from most impactful to least impactful based on their contribution to the prediction [17,49]. The negative values on the X-axis indicate a lower risk prediction, whereas the positive values on the right side indicate a higher risk prediction. The color represents the feature value; blue indicates the low feature value, and red indicates the high feature value. In the scenario with the XGboost classifier and RF-voting ensemble, the AO feature was placed in the first rank, indicating the feature is at a high risk of CVD. The features rank can be seen in Figure 4a,b. Figure 4a shows a more spread-out distribution, indicating that multiple features contribute to the model’s decision. Despite using more features, XGBoost may not effectively generalize due to feature interactions or overfitting.

Table 11 shows that combining the individual supervised learning model with the voting ensemble increases the model’s performance. The most significant improvement, almost 12%, occurred in the comparison of individual RF performance with the RF-voting ensemble. When combined with the voting ensemble, the individual CATBoost model and the LightGBM model experienced an increase of 2.5%, while other models only experienced an increase of 3%. Essentially, all the models showed improvement.

## 4. Discussion

The initial stage of this study involved data cleaning. Based on the number identification (ID) of patients in the repository, the 563 ultrasound data from the diagnosis result and treatment sections were compared with the 36,994 ultrasound data from the patients’ visitation history section. During data cleaning, we checked the columns for completeness and appropriate data types, categorizing columns as either numeric or categorical. Incomplete entries were handled by replacing null values with the median of each column. The gender column, containing categorical values, was encoded by assigning 1 to “M” instances and 0 to “F” instances. Next, an event-based self-similarity approach was used to identify patterns within time-series by comparing similar events across different instances in grouping patients. The term even refers to a significant change in echocardiographic characteristics. By quantifying self-similarity between events using euclidean distance metrics, unique patterns were detected, indicating high-risk patients. After preprocessing, the data was split into two samples (sample A and sample B). Each part was grouped into nine cohorts. Descriptive statistics were then applied to observe the data distribution of different clusters.

The normalized and unnormalized techniques are implemented to analyze the impact of the model’s performance. The subsequent data normalization processes applied the Z-score normalization technique and absolute maximum normalization technique to enhance data consistency and reduce the risk of overfitting. This normalization minimized dimensionality issues and bolstered dataset reliability for robust model training.

The model development framework entailed an exploratory search for optimal supervised learning models, each trained separately on samples A and B. Each model’s hyperparameters were fine-tuned for optimal performance. XGBoost builds trees sequentially to correct previous errors. XGBoost was configured with 100 estimators, considering the balance between accuracy and computational efficiency. Too few estimators can cause underfitting, while too many estimators can increase training time and the risk of overfitting. The learning rate controls how much each tree contributes to the final prediction. If the learning rate is below 0.3, more trees are needed for convergence, which results in increased computation time. The proposed XGBoost’s learning rate of 0.3 was moderate, allowing the model to learn effectively without requiring an excessive number of trees. On the other hand, RF builds trees independently and averages predictions to reduce variance. RF was set with nine estimators to keep computational costs low while maintaining sufficient model diversity for accurate predictions, whereas a random state of 1 to ensures the results’ reproducibility. CATBoost was set to a depth of 6 to ensure the balancing model interpretability and predictive power while keeping training efficient. Underfitting may occur when shallow trees have a depth below 4. Ensuring enough boosting steps for improved accuracy without excessive computation time, the iteration of CATBoost was up to 100 times per tree. Contrary to XGBoost, the LightGBM preferred a lower learning rate because it is more aggresive in growing trees. For LightGBM’s learning rate parameter, a lower learning rate allows for gradual optimization, reducing overfitting. The proposed LightGBM employed 31 leaves per tree with a learning rate of 0.1 for balancing generalization and learning capacity. Each model’s performance was assessed based on accuracy, precision, recall, F1-score. In addition, Cohen’s Kappa was utilized in this study to measure the interrater agreement. The Cohen’s Kappa metrics is frequently employed in classification problems to measure model performance in datasets with imbalanced values [50,51]. For further validation, 10-fold cross-validation was performed in sample B, achieving mean accuracy scores above 85% for all models.

**Figure 5:**
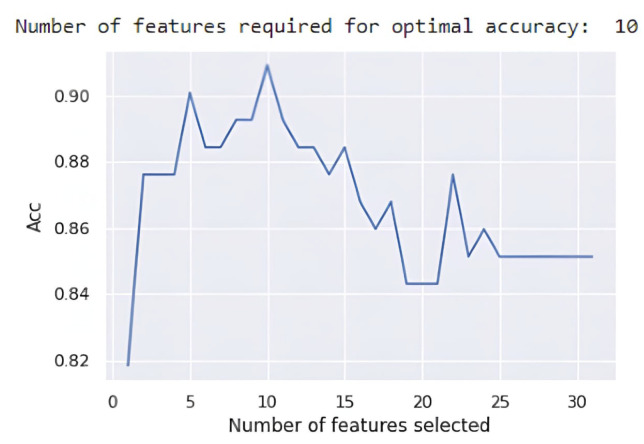
RFE technique implementation.

Several features can help to identify the treatment and predict future diagnosis after treatment. Incorporating the supervised learning models XGBoost, RF, LightGBM, and CATBoost with the RFE technique facilitated the examination of possible feature combinations. The feature selection process was conducted using the RFE technique in Python 3.7.15. Figure 5 shows the compilation result of the RFE technique. The Explainable AI (XAI) technique offers an effective method for analyzing the importance of features within model predictive outcomes [49]. The importance of features was assessed using the SHAP method, which is one of the XAI. Key features influencing treatment outcomes and diagnostic predictions included age, AO, LV, and LA. These features, particularly impactful in the RF ensemble classifier, were consistent with prior findings, such as those from Wang [1], which identified age as an essential feature. These echocardiogram-based measurements provide clinicians with valuable diagnostic and prognostic insights. For example, in Group 23, we identified parameters associated with more prolonged survival among ventricular fibrillation patients. In contrast, Group 31 patients could benefit from more aggressive interventions, including defibrillator implantation or surgical treatments. While this study’s dataset size is limited, increasing the data volume in future work could expand the clinical utility of machine learning to guide treatment planning based on ultrasound results.

**Figure 6:**
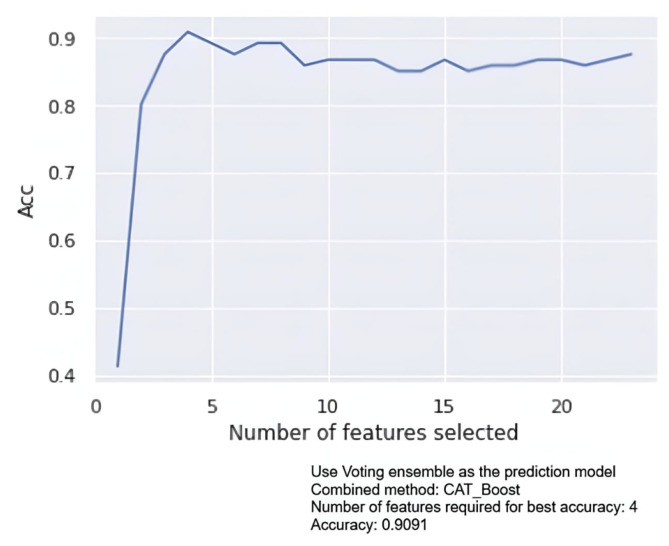
Voting ensemble with CATBoost implementation.

Afterward, to enhance the model’s performance, the single model of supervised learning framework was combined using a voting ensemble method and was employed in the dataset sample B. In this second framework, multi-model approaches can leverage the strengths of multiple models and reduce any single model’s bias. Multi-model approaches can also be made more robust and resistant to noise in the data. Figure 6 displays the results of executing the voting ensemble method with a single model in the form of a graph. The graph highlights the correspondence between the accuracy value and the number of features, the model name combined with voting, the accuracy value achieved when voting is combined, and the number of features supporting this accuracy.

**Figure 7:**
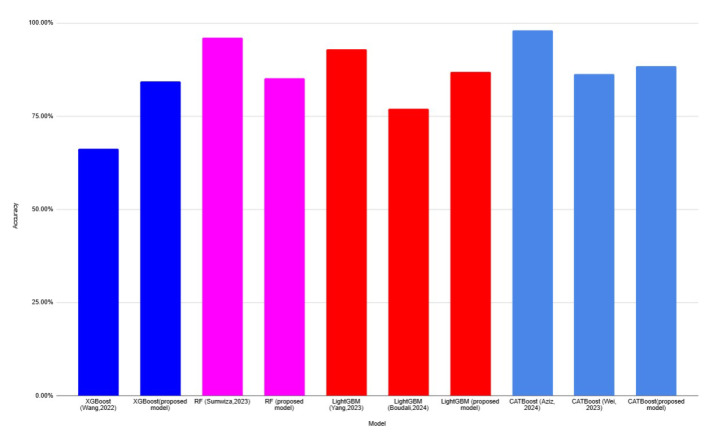
Comparison of the proposed study with the previous studies.

**Figure 8:**
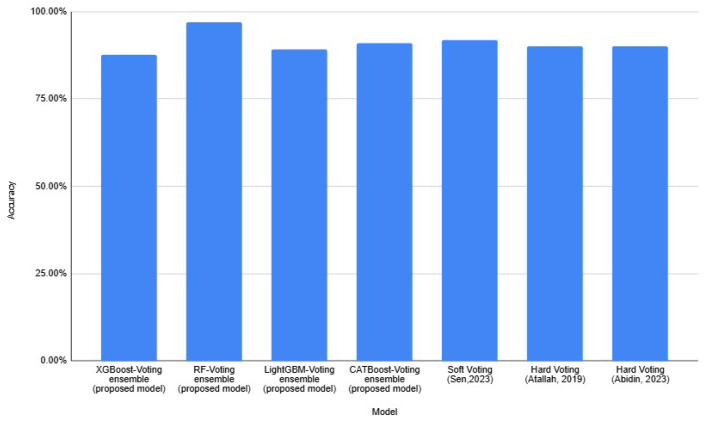
Comparison of the proposed voting ensemble model with the previous studies.

Table 12 shows the comparison of accuracy between individual models, voting ensemble models, and previous studies. Figure 7 depicts the accuracy comparable between existing models and the proposed models. The proposed models have consistently high accuracy, above approximately 80%, indicating the proposed approaches are effective. The proposed models outperformed the earlier models by [1,32]. The performances of XGBoost and LightGBM from earlier studies show significantly lower accuracy. An accuracy of 66.30% was achieved when using XGBoost [1], whereas LightGBM [32] reached its accuracy at 77%. In [28], the RF model was employed, resulting in a 96% accuracy. Yang [29] demonstrated that LightGBM achieved an accuracy of 93%, whereas [33,34] reported the CATBoost’s accuracy scores of 86.30% and 98.08%, respectively. Some existing models appear comparable or even slightly higher than the proposed models, implying that the proposed models are still competitive. Figure 8 shows that each proposed voting ensemble model achieves a high accuracy, exceeding 85%, indicating strong predictive performance. The performance of the proposed RF-voting ensemble outperformed the model’s performance, which was studied by [35]. Using voting ensemble learning improves generalization, and its effectiveness in CVD prognosis remains promising and competitve with state-of-the-art models.

## 5. Conclusions and Future Work

Immersive technologies impact the development of data formats. Machine learning techniques can categorize types of heart disease by analyzing a patient’s echocardiographic data. This study demonstrated the utilizing event-based self-similarity for the effectiveness of machine learning in the automated prognosis of CVD, addressing the challenges posed by imbalanced echocardiogram data. The results indicate that class imbalance significantly affected model performance, with CATBoost model yielded the best individual results. However, ensemble techniques, particularly the voting ensemble approach, provided a more a robust solution, achieving an accuracy of 96.9% when combined with the RF model. This highlights the realibility of the ensemble voting approach in improving predictive performance. Furthermore, key features—age, AO, LV, and LA—were identified as significant predictors for CVD prognosis, reinforcing the model’s practical applicability in aiding physicians with early diagnosis and personalized treatment strategies. Even though the RFE technique was effective for future works, other feature selection methods, such as LASSO regression, SHAP values, or hybrid techniques, could be explored to enhance model interpretability and accuracy further. To address the class imbalance issue, the data augmentation approach, such as synthetic minority over-sampling, can be used on minority classes. For further advance, the deep learning method can also be complemented with an automation prognosis system. Overall, these findings highlight the potential of automated prognosis systems to assist physicians, with machine learning offering further improvements for personalized treatment of CVD patients, especially in the context of imbalanced datasets. By refining feature selection, optimizing class balance strategies, and leveraging advanced learning techniques. These findings pave the way for future advancements in AI-driven healthcare technologies.

## Figures and Tables

**Table 1:** Some recent studies on CVD.

Authors	Best Model	Accuracy	Total Parameter	Total Samples	Repository
Wang [1]	XGBoost	66.3%	30	5360	MIMIC-III
Sachdeva [19]	SVM	96.67%	13	299	UCI Heart Failure Clinical
Sumwiza [28]	RF	96%	14	1025	Kaggle
Yang [29]	LightGBM	93%	10	4240	CHD Framingham Heart Institute
Boudali [32]	LightGBM	77%	21	47,786	BRFSS
Aziz [33]	CATBoost	98.08%	14	n.a	UCI Cleveland
Wei [34]	CATBoost	86.30%	11	918	Kaggle
Sen [35]	Soft voting	91.85%	11	918	UCI and Statlog
Atallah [36]	Hard voting ensemble	90%	14	303	UCI
Abidin [37]	Hard voting ensemble	90%	11	303	Kaggle

**Table 2:** Grouping patient treatments and diagnosis categories.

Treatments in Three-Year Follow-Up	Died	Follow-Up	No Follow-Up
Cardiac catheter ablation	Group 11	Group 13	Group 14
Ventricular defibrillators	Group 21	Group 23	Group 24
Drug control	Group 31	Group 33	Group 34

**Table 3:** The Sample A’s distribution in diagnosis and treatment categories.

Treatments in Three-Year Follow-Up	Sample A		
	Died	Follow-Up	No Follow-Up
Cardiac Catheter Ablation	6	37	5
Ventricular Defibrillator	66	220	77
Drug Control	10	116	10

**Table 4:** The Sample B’s distribution in diagnosis and treatment categories.

Treatments in Three-Year Follow-Up	Sample B		
	Died	Follow-Up	No Follow-Up
Cardiac Catheter Ablation	20	100	22
Ventricular Defibrillator	150	498	121
Drug Control	24	260	17

**Table 5:** The descriptive statistics result of group 23.

Group23	Age	LV (mm)	VS (mm)	LVPW (mm)	LA (mm)	AO (mm)	TR_PG_Mean (mmHG)	LVEF
count	220	220	220	220	220	220	220	220
mean	76.7	46.8	13	12.6	46.5	36.2	24.2	56
std	13.3	7.5	2.1	1.6	9.6	4.5	8.1	8.1
min	26	4	8	7	0	24	10	21
25%	69	42	11.7	11.4	41	33	18	52
50%	81	45.7	13	12.4	45.4	36	23	57
75%	86	51.4	14	13.9	51.6	39	27.8	61
max	96	75	21	18.1	100	51	57	69

**Table 6:** Supervised techniques accuracy.

Models	Sample A			Sample B		
	Raw Dataset	Z-Score	Abs Maximum	Raw Dataset	Z-Score	Abs Maximum
XGBoost	0.406	0.406	0.406	0.843	0.843	0.843
RF	0.406	0.406	0.406	0.851	0.868	0.851
LightGBM	0.394	0.412	0.394	0.868	0.876	0.868
CATBoost	0.394	0.394	0.394	0.884	0.884	0.884

**Table 7:** The results of train evaluation metrics.

Model	Precision	Recall	F1-Score	Cohen’s Kappa
XGBoost	0.992	0.992	0.992	0.984
RF	1.000	1.000	1.000	1.000
LightGBM	0.990	0.990	0.990	0.980
CATBoost	0.992	0.992	0.992	0.984

**Table 8:** The results of testing evaluation metrics.

Model	Precision	Recall	F1-Score	Cohen’s Kappa
XGBoost	0.949	0.917	0.932	0.874
RF	0.958	0.959	0.955	0.913
LightGBM	0.958	0.942	0.950	0.905
CATBoost	0.990	0.990	0.989	0.980

**Table 9:** The K-fold cross validation results.

K-Fold	XGBoost	RF	LightGBM	CATBoost
1	0.913	0.835	0.913	0.874
2	0.874	0.850	0.898	0.874
3	0.913	0.882	0.921	0.889
4	0.882	0.866	0.906	0.889
5	0.850	0.819	0.843	0.835
6	0.866	0.858	0.874	0.866
7	0.906	0.866	0.882	0.889
8	0.889	0.850	0.906	0.850
9	0.850	0.819	0.898	0.835
10	0.865	0.881	0.881	0.857

**Table 10:** The accuracy (A) of comparison models (ensemble technique) with different number of features (F).

Predictive Models Features	XGBoost		RF		LightGBM		CATBoost	
	A	F	A	F	A	F	A	F
XGBoost	0.851	12	0.876	8	0.893	7	0.893	5
RF	0.884	8	0.876	4	0.893	7	0.893	4
LightGBM	0.876	15	0.901	8	0.901	7	0.901	7
CATBoost	0.868	11	0.893	9	0.893	9	0.884	7
Voting Ensemble	0.876	8	0.969	4	0.893	7	0.909	4

**Table 11:** The comparison accuracy between individual models and voting ensemble model.

Model	Individual	Voting Ensemble
XGBoost	0.843	0.876
RF	0.851	0.969
LightGBM	0.868	0.893
CATBoost	0.884	0.909

**Table 12:** The comparison of accuracy between individual models, voting ensemble models, and previous studies.

Model	Accuracy
XGBoost [1]	0.663
RF [28]	0.960
LightGBM [29]	0.930
LightGBM [32]	0.770
CATBoost [33]	0.980
CATBoost [34]	0.863
Soft voting [35]	0.918
Hard voting [36]	0.900
Hard voting [37]	0.900
XGBoost proposed model	0.843
RF proposed model	0.851
LightGBM proposed model	0.868
CATBoost proposed model	0.884
XGBoost-Voting ensemble proposed model	0.876
RF-Voting ensemble proposed model	0.969
LightGBM-Voting ensemble proposed model	0.893
CATBoost-Voting ensemble proposed model	0.909

**Figure A1:**
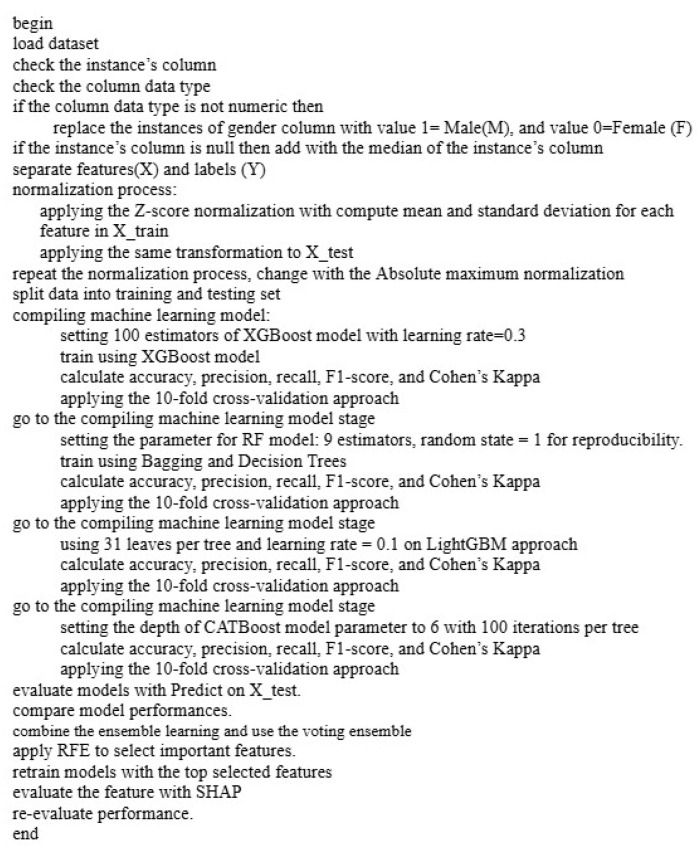
Pseudocode of proposed model.

## Data Availability

The data that support the findings of this study are not openly available due to reasons of sensitivity and are available from the corresponding author upon reasonable request. Data are located in controlled access data storage at the Department of Cardiology, Taichung Veterans General Hospital.
